# The role of enterprise architecture-driven dynamic capabilities and operational digital ambidexterity in driving business value under the COVID-19 shock

**DOI:** 10.1016/j.heliyon.2022.e11484

**Published:** 2022-11-08

**Authors:** Rogier van de Wetering

**Affiliations:** Faculty of Science, Department of Information Sciences, The Open University, the Netherlands

**Keywords:** Dynamic capabilities, Enterprise architecture (EA), EA-Driven dynamic capabilities, Digital dynamic capability, Operational digital ambidexterity, Business value under COVID-19

## Abstract

This article builds upon the dynamic capabilities view and argues that firms that developed their dynamic capabilities using enterprise architecture (EA) are better equipped to cope with the COVID-19 shock. An online survey collected data from 414 senior practitioners, business and IT managers, and executives. The study’s research model containing three hypotheses was assessed by applying Partial Least Squares (PLS) structural equation modeling (SEM), PLS-SEM. Outcomes point out that dynamic capabilities driven by EA enhance the firm’s digital dynamic capability and, therefore, the competencies to manage digital technologies. This capability subsequently enhances the firms' operational digital ambidexterity. Also, outcomes show that operational digital ambidexterity significantly impacts business value. This study advances our knowledge and insights on developing EA-driven dynamic capabilities under COVID-19 and unfolds key areas where decision-makers should invest in enhancing business value.

## 1 Introduction

The Coronavirus disease (COVID-19) has affected businesses, societies, countries, and territories worldwide in an unprecedented way. This pandemic has practically changed how people live and interact due to social distancing. Also, we saw increased uptake of SMAC technologies (i.e., social media, mobile apps, data analytics, and cloud-based solutions) to accelerate the transformation of remote workforce solutions and service delivery and interaction models to address customers' and employees' needs and wishes (Li et al., 2022; Rakshit et al., 2021).

The acute conditions of COVID-19 imposed many complex challenges for firms. For instance, COVID-19 has led to great uncertainty and an involuntary shift in consumer and b2b wishes, needs, and behavior (Markovic et al., 2021). Moreover, various scholars argue that under these severe conditions, firms must develop adaptive and digital capabilities to leverage digital technologies and meet customers' needs and wishes adequately in an increasingly complex business environment (Heredia et al., 2022; Ozanne et al., 2022; Sadreddin and Chan, 2022). Hence, under these turbulent conditions, firms need to be adaptive and flexible to adequately deploy their business and Information Technology (IT) resources and capabilities in response to emerging disruptions and reconfigure the business processes, business and service delivery models accordingly (Clauss et al., 2022; Coreynen et al., 2017; Klein and Todesco, 2021; Miroshnychenko et al., 2021; Randhawa et al., 2021; Van Looy, 2021).

In this process, modern firms use Enterprise Architecture (EA). EA can be considered a blueprint of firms that helps orchestrate the IT systems portfolio (including digital platforms, cloud solutions, data centers, business intelligence, and AI), business processes, and capabilities and their interrelationships (Grave et al., 2021; Shanks et al., 2018). As can be gleaned from this description, EA does not only entail IT but focuses on bridging communication gaps between business and IT stakeholders and offers firms many other benefits (Kotusev et al., 2022; Pattij et al., 2022; van de Wetering et al., 2021a, 2021b; Zhang et al., 2018). In doing so, EA, e.g., leverages IT so business processes can be appropriately executed in line with the firm’s strategic priorities using individual artifacts (Kotusev et al., 2022; Zhang et al., 2018). These artifacts are related to general principles, maxims, considerations, and detailed designs of full solution architectures and IT landscapes, including technology blueprints and improvement roadmaps (Grave et al., 2021; Kotusev et al., 2022). Thus, EA is an essential practice for the firm’s design and orchestrating and restructuring of the organization’s IT and business resources when needed (Leih et al., 2015; Moscoso-Zea et al., 2019). Moreover, especially in rapidly changing business ecosystems, EA continuously guides the alignment of the IT strategy and portfolio, and business landscape and drives digital innovation and transformations to create value for the business and its key stakeholders (Korhonen et al., 2016; Niemi and Pekkola, 2019; Pattij et al., 2022; Ross et al., 2019; Törmer and Henningsson, 2019a; van de Wetering, 2021; van de Wetering et al., 2021a, 2021b).

Over the past few years, the information systems (IS) discipline embraced the dynamic capabilities view (DCV) to unfold and show the business value of EA. In particular, scholars have focused on the concept of EA-driven capabilities for adaptiveness, innovativeness, and digital transformation of the firm (Korhonen and Halén, 2017; Korhonen et al., 2016; Pattij et al., 2022; Shanks et al., 2018; Törmer and Henningsson, 2019b; Van de Wetering, 2019; van de Wetering, 2021). EA-driven capabilities can be considered the ability of firms to “…orchestrate and employ the firm’s resources using EA while simultaneously trying to align strategic goals, and objectives with the usage of IS/IT” (Van de Wetering, 2019, p. 1). Previous work conceptualized these EA-driven capabilities as dynamic capabilities (Grave et al., 2021; Shanks et al., 2018; Törmer and Henningsson, 2019b; van de Wetering et al., 2021a, 2021b; van de Wetering et al., 2020) arguing that they equip firms with the ability to sense and capitalize upon business and digital innovation opportunities and reconfigure business processes accordingly (Shanks et al., 2018; van de Wetering, 2021). Therefore, these capabilities can be considered the firm’s ability to use and leverage EA to sense new business processes and digital innovations, and strategic threats, mobilize the firm’s resources and transform business operations using digital technologies to address the rapidly changing business and organizational environment (van de Wetering et al., 2021a, 2021b).

Some scholars argue that these capabilities are critical antecedents of day-to-day activities and support current products and services, i.e., operational capabilities (Van de Wetering, 2019). This is consistent with the observations of Van den Berg et al. (2019) and Ahlemann et al. (2021) that showcased the intermediating role of EA-enabled outcomes in the nomological value path. However, recent studies question the depth of which these benefit-realization and value-creating mechanisms are fully understood by the literature (Ahlemann et al., 2021; Tamm et al., 2022) and, therefore, less clarity remains “..around how organizations should go about achieving these benefits (Tamm et al., 2022, p. 2). Moreover, despite high expectations concerning EA and EA-driven capabilities, many EA initiatives in practice fail or do not live up to stakeholder expectations indicating that it is still not fully understood how EA-driven capabilities shape digital innovations and transformation in practice (Al-Kharusi et al., 2021; Ross et al., 2019; Törmer and Henningsson, 2019a). As firms across industries have to deal with hyper-competition also driven by technological advancements, EA-driven capabilities must be at the center of strategic and digital innovation planning in enabling flexibility and agility and adapting and aligning the firm’s resources to changing customer needs and demands (Ross et al., 2019; Törmer and Henningsson, 2019b; van de Wetering, 2021).

Thus, despite the valuable contributions of the extant literature, there is currently an inadequate understanding of how EA, and in particular EA-driven capabilities, facilitate operational digital innovation and, thus, the ability to continually innovate and improve the firm’s operational processes using digital technologies (i.e., operational digital ambidexterity), and deliver business value under the extraordinary circumstances (Lee et al., 2015; Magnusson et al., 2020; Ozanne et al., 2022; Sadreddin and Chan, 2022). Moreover, there is a scant scholarship that focuses on practitioner recommendations. Therefore, the literature is fragmented, and our current understanding of the role and impact of EA-driven capabilities during turbulent conditions of the COVID-19 shock remains limited. Thus, notable gaps remain in the literature. However, gaining these insights is crucial because firms need to have the capabilities to adequately leverage digital technologies and competencies during crises like the COVID-19 pandemic (Khurana et al., 2022; Sadreddin and Chan, 2022).

Therefore, it is crucial to investigate the mechanisms of how firms could genuinely leverage their EA-driven dynamic capabilities and digital investments and create business value.

This article claims that firms that have developed their EA-driven dynamic capabilities can handle and proactively address the exogenous COVID-19 shock and adjust accordingly. Furthermore, consistent with the dynamic capabilities theory (Fainshmidt et al., 2019; Lin and Wu, 2014; Teece, 2007), these firms are likely to be operational ambidextrous in their ability to innovate continually and improve their operational processes using digital technologies and enhance IT-enabled business value (Heredia et al., 2022; Lee et al., 2015; Sadreddin and Chan, 2022).

The dynamic capabilities view (DCV) is considered an appropriate starting point for further investigation. It considers the targeted use and deployment of the firm assets and resources, including digital innovations, as a differentiating and value-creating force within organizations (Khalil and Belitski, 2020; Marx et al., 2021) under different environmental and market conditions (Fainshmidt et al., 2016).

Specifically, this paper examines if EA-driven dynamic capabilities influence the firms' digital dynamic capability, i.e., the qualities and competencies that a firm possesses to develop and manage innovative digital technologies (Khin and Ho, 2019). This technical-oriented capability is crucial when firms want to use data and artificial intelligence (AI) driven innovations in practice, for instance, to reinforce existing operations and develop digital-driven operational processes and services and efficient ways of working (Chen et al., 2021; Dwivedi et al., 2019; Gupta et al., 2020; Mittal, 2020).

Second, this study investigates if the firm’s process-based ability to continually innovate and improve its operational processes using digital technologies through an exploratory mode and an exploitation mode and thus a dual approach to digitalization, i.e., operational digital ambidexterity, facilitated by digital dynamic capability, will lead to business value under COVID-19. These study outcomes collectively should reveal the value-creating mechanisms of EA-driven dynamic capabilities during COVID-19.

Therefore, given the above, this work will address the following research question: Through which mechanism can firms gain business value under the COVID-19 shock from their EA-driven dynamic capabilities?

This study builds a research model with associated hypotheses in addressing this question. It empirically validates this model using a large-scale, cross-sectional survey of 414 decision-makers and senior practitioners, e.g., Chief executive officer (CEO), Chief information officer (CIO), Chief digital officer (CDO), IT and business management, and lead enterprise architects.

Notable results are that dynamic capabilities driven by EA enable firms' digital dynamic capability that subsequently improves the firm’s operational digital ambidexterity. Moreover, operational digital ambidexterity significantly impacts business value under COVID-19. This work extends the literature by revealing the more comprehensive yet rich mechanisms between dynamic capabilities and business value under the COVID-19 shock. These contributions are also significant concerning the IS discipline’s role in addressing the great uncertainty and today’s many complex challenges (Sarker et al., 2019).

The following section outlines the relevant theoretical lenses and the hypotheses development. The subsequent sections present the methods, the study outcomes, and the discussion. Finally, this study ends with concluding remarks.

## 2 Literature review

### 2.1 The dynamic capabilities view and enterprise architecture’s role

The DCV draws on multiple disciplines and mainly builds upon the resource-based view of the firm (RBV) (Peteraf, 1993). The RBV explains how firms can sustain competitiveness using their IT and business resources (Jeffers et al., 2008). The extant literature distinguishes between utilizing the firm’s core resources and the capability development process. Both can be considered the primary elements of resources-orchestration theories (Burin et al., 2020; Donada et al., 2016; Luo, 2002). Firm resources can be regarded as the assets that a firm possesses or can control for that matter (Jeffers et al., 2008). Capabilities are firm-specific and deploy the resources (e.g., IT or EA) to achieve a particular strategic goal or ambition (Soto-Acosta, 2020). Many scholars consider the DCV a leading strategic and theoretical framework (Fainshmidt et al., 2019; Inan and Bititci, 2015; Loureiro et al., 2021). Dynamic capabilities can be considered the stance and orientation to persistently integrate, reconfigure and renew the firm’s resource base and capabilities to address market disruptions and changes (Ferreira et al., 2020; Lin and Wu, 2014; Wang and Ahmed, 2007). Dynamic capabilities enable firms to sense business opportunities, mobilize resources in line with these opportunities and transform and renew the organization to anticipate market disruptions and business changes (Drnevich and Kriauciunas, 2011; Schmidt and Scaringella, 2020). These adaptive capabilities must be considered while deploying the firm’s business and innovation strategy (Chirumalla, 2021; Eshima and Anderson, 2017; Loureiro et al., 2021; Wetering, 2022).

Within the DCV, a clear distinction can be made between dynamic capabilities and their operational counterpart (Barrales-Molina et al., 2015; Protogerou et al., 2012; Zollo and Winter, 1999). The former encompasses the firm’s ability to change and execute under turbulent conditions, like the COVID-19 pandemic, and supports the firm’s resilience and innovative stance (Dejardin et al., 2022; Ozanne et al., 2022). On the other hand, operational capabilities relate to the present and focus on producing and delivering products and influencing business value (Moreno et al., 2020). Therefore, it is clear that dynamic capabilities do not directly impact business value but do so indirectly through the firms' operational capabilities (Moreno et al., 2020; Protogerou et al., 2012).

To develop dynamic capabilities, firms must actively invest in EA (Ross et al., 2019; Törmer and Henningsson, 2019b; van de Wetering, 2021). EA facilitates structuring IT technology components supporting the business and its adaptive capacities. EA, therefore, considers multiple layered representations, such as business operations, end-to-end processes, information, services, and technical infrastructure. Several recent studies showed that dynamic capabilities, driven by EA, support firms in decision-making, IT orchestration, and digital capability development (Grave et al., 2021; Hafsi and Assar, 2016; Korhonen et al., 2016; Shanks et al., 2018; van de Wetering, 2021). These particular capabilities allow firms to use EA as a guide to share the firm’s assets and recompose and renew the organization’s resources to shape and orient themselves in line with ever-changing environments (Gomes, 2015; Shanks et al., 2018; Törmer and Henningsson, 2019b; Van de Wetering, 2019). These studies argue that firms need to infuse EA into the firm’s adaptive capabilities and routines to achieve business value with EA. These capabilities actively use EA to sense and identify business possibilities and even possible threats and subsequently orchestrate firm resources to capitalize upon the possibilities in conjunction with the firm’s strategies by engaging the business and IT stakeholders (Penttinen and Isomäki, 2010; Shanks et al., 2018; van de Wetering et al., 2021a, 2021b).

In summary, EA can drive significant business benefits. These benefits include strategic planning, increased business and IT integration and alignment, cost reduction through portfolio rationalization, efficient business processes, revenue enhancements, and the firm’s ability to bring products and services to the market earlier (Ahlemann et al., 2021; Grave et al., 2021; Niemi and Pekkola, 2019). In addition, pressing regulatory changes, the emergence of new business models, and changing market drivers require fast business and IT landscape adaptation (Törmer and Henningsson, 2019a). When firms cannot drive new transformation initiatives based upon EA, they will highly likely, end up having fragmented process support with a loss of end-to-end view, a highly complex, costly, non-transparent, and redundant IT portfolio without linkage to business strategies and goals, resulting in the inability to respond to market trends (Al-Kharusi et al., 2021; Niemi, 2008; Törmer and Henningsson, 2019b; Van den Berg et al., 2019). Therefore, the EA benefits can only be realized by ensuring that EA is infused into the firm’s dynamic capabilities to act and respond to organizational and environmental changes (Grave et al., 2021; Ross et al., 2019; Törmer and Henningsson, 2019a; van de Wetering, 2021; van de Wetering et al., 2021a, 2021b).

### 2.2 Digital dynamic capability and the ambidexterity perspective.

Digital dynamic capability is a key technical capability that can be regarded as the firm’s technical talent, competencies, and the organization’s skill, talent, and business sense to proactively govern and manage digital technologies (Chaudhuri et al., 2022; Khin and Ho, 2019; Nousopoulou et al., 2022). Such a digital capability is crucial under the severe conditions driven by COVID-19, as it allows firms to use adequately identify and obtain technical skills and competencies and master digital technologies that are needed to enhance business processes so that the firms will outperform others during the COVID-19 crisis (Li et al., 2022).

These digital technologies include distributed ledger technologies, big data analytics, social media, mobile technologies, cloud, and AI. Therefore, this technical-oriented dynamic capability can be understood as the firms' ability to manage and master digital technologies and develop innovative, data-driven services and products that drive digital transformations.

In this regard, innovations enabled by digital technologies can be referred to as digital innovation (Chirumalla, 2021; Ciriello et al., 2018; Nambisan et al., 2017) and are often disruptive (Christensen et al., 2003).

This study embraces a hierarchical capability view and conceptualizes digital dynamic capability as a lower-order technical-oriented capability (Božič and Dimovski, 2019; Lee et al., 2015; Van de Wetering and Versendaal, 2021). Higher-order dynamic capabilities drive these capabilities (Wang and Ahmed, 2007), in this case, EA-driven dynamic capabilities. It is required to make substantial undertakings to embrace new digital technologies (Khin and Ho, 2019; Wu et al., 2019) to develop this capability, requiring specific, inimitable resources and skills (Chaudhuri et al., 2022; Heredia et al., 2022).

Digital- and data-driven innovation is a major challenge for modern firms (Dwivedi et al., 2019; Li et al., 2022; Sadreddin and Chan, 2022; Schmidt and Scaringella, 2020). It is a true challenge, as firms need to deploy and enable their IT assets, skills, and resources optimally, and also align their organizational, IT, and knowledge capabilities to be innovative and hence to efficaciously respond to inherent changes in industry and market conditions faster than competitors (Chirumalla, 2021; Ferreira et al., 2020). The literature has identified two unique mechanisms for firms to leverage their resources and capabilities to foster innovation: exploration and exploitation (Faridian and Neubaum, 2021; Jansen, Tempelaar, Van den Bosch and Volberda, 2009; March, 1991). Exploration concerns the firm’s efforts to invent new operational techniques or operational processes and fundamentally change or invent new business operations (e.g., product/service development and production, supply chain management, customer delivery, and employee management) using digital technology. On the other hand, exploration focuses on enhancing operational productivity by improving current operations' efficiency and cycle time and reducing costs using digital technology (Lee et al., 2015). The extant literature refers to this simultaneous pursuit of exploration and exploitation as ‘ambidexterity’ (Jansen et al., 2012; Raisch et al., 2009). Therefore, this study follows Lee et al. (2015, p. 401) and defines operational digital ambidexterity as a firm’s ability to simultaneously pursue operational digital exploration and exploitation. It thus refers to the ability of a firm to innovate and improve its operational processes using digital technologies. Organizations' simultaneous execution of the opposing modes (exploration vs. exploitation) will offer them competitiveness (Faridian and Neubaum, 2021; Luger et al., 2018; Raisch et al., 2009).

## 3 Theoretical context and hypotheses development

**Figure 1:**
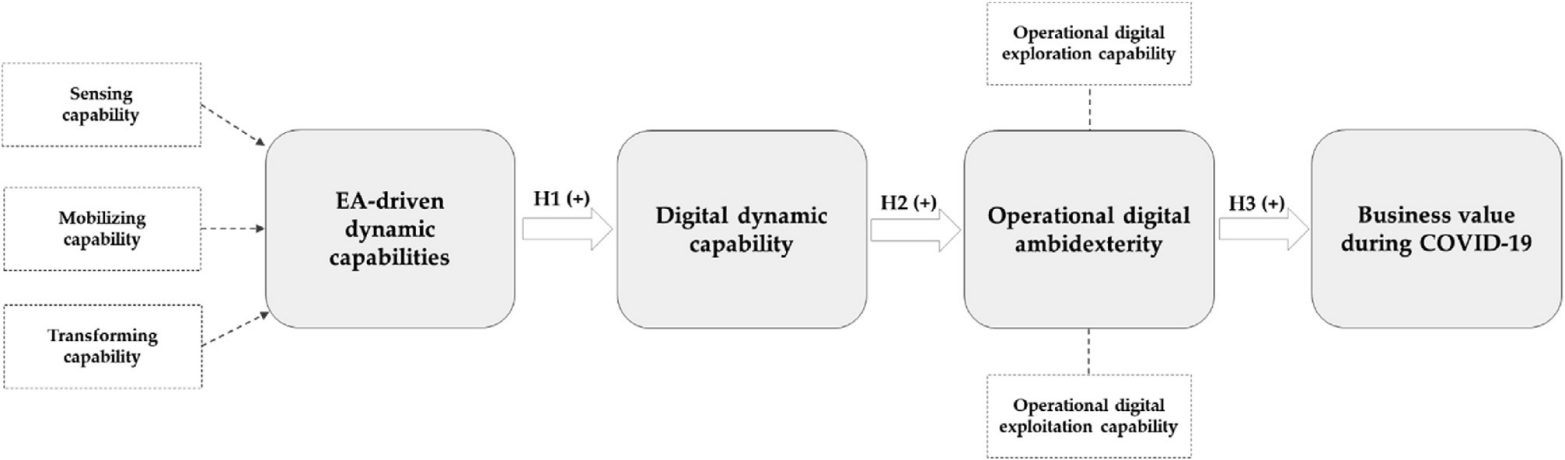
Research model.

This study’s conceptual model is shown in Figure 1 and highlights four important constructs, EA-driven dynamic capabilities (i.e., sensing, mobilizing, and transforming), digital dynamic capability, operational digital ambidexterity (i.e., operational digital exploration and exploitation capability), and business value under COVID-19. In addition, this study identifies essential intermediate-process-based digital abilities and organizational mechanisms consistent with previous dynamic capability literature, showcasing the indirect effect of dynamic capabilities on business value (Heredia et al., 2022; Makkonen et al., 2014; Protogerou et al., 2012; Sadreddin and Chan, 2022). Table 1 summarizes the research model’s constructs and definitions.

**Table 1:** The **r**esearch model’s constructs description.

Construct	Description	Literature
EA-driven dynamic capabilities	The ability of firms to use EA in the process of sensing new business processes and digital innovations, and strategic threats, mobilizing the firm’s resources and seizing opportunities when they present themselves, and transforming business operations using digital technologies and realigning its workforce to address the rapidly changing business and organizational environment.	(van de Wetering, 2021).
Digital dynamic capability	Represents qualities and competencies required to manage innovative digital technologies for new exceptional and effective service development.	(Khin and Ho, 2019)
Operational digital ambidexterity	The process-based ability to continually innovate and improve the firm’s operational processes using digital technologies and capabilities through an exploratory and exploitation mode and, thus, a dual digitalization approach.	(Lee et al., 2015; Magnusson et al., 2020)
Business value under COVID-19	The value created through the firm capabilities' as measured by product quality, efficiency, and level of customization during COVID-19.	(Hsu, 2013; Rodrigues et al., 2020; Van de Wetering, 2019)

### 3.1 EA-driven dynamic capabilities and digital dynamic capability

Consistent with the DCV and EA-driven capabilities scholarship, recent literature has identified and empirically validated three unique EA-driven dynamic capabilities (Van de Wetering, 2019). These EA-driven dynamic capabilities are ‘Sensing,’ ‘Mobilizing,’ and ‘Transforming.’ EA-driven sensing capability equips firms with the ability to spot, interpret, and pursue new digital innovations, like IoT, big data, AI, and cloud. It also allows firms to identify business, process opportunities, and strategic threats (Törmer and Henningsson, 2019a; van de Wetering, 2021). This capability also strengthens EA resources' deployment to enhance business operations while maintaining close alignment with the firms' stakeholders' business demands, needs, and wishes (van de Wetering et al., 2020). A mobilizing capability allows a firm to seize opportunities when they present themselves (Teece, 2007; Vanpoucke et al., 2014). This capability orchestrates portfolio investments and the selection process of improvement projects that drive the firm’s digital transformation (Shanks et al., 2018; Törmer and Henningsson, 2019b; van de Wetering et al., 2020). Finally, a transforming capability uses the firm’s EA and resource base to redesign operational procedures and business operations using digital technologies and re-align its workforce (Drnevich and Kriauciunas, 2011; Shanks et al., 2018; van de Wetering, 2021). A transforming capability drives the firm’s flexible adaptation of human resources and aligns stakeholders' interests with the business (Korhonen and Halén, 2017; Protogerou et al., 2012; Van de Wetering, 2019).

EA-driven dynamic capabilities support firms in optimizing operational digital ambidexterity by facilitating diverse processes and capabilities. One of those critical capabilities is a digital dynamic capability. The extant literature has shown that the competitiveness of firms stems from the optimal deployment of the business and IT resources and driving digital capabilities (Gong and Ribiere, 2021; Ma et al., 2021; Moreno et al., 2020; Ross et al., 2019). Also, strategic investments in the firm’s digital resources and capabilities are essential to developing organizational capabilities and driving IT business value (Chirumalla, 2021). Firms can leverage their EA-driven dynamic capabilities to shape their digital capability further and, thus, their competence in orchestrating and mobilizing innovative digital technologies to drive innovative practices (Denner et al., 2018; Grave et al., 2021; Shanks et al., 2018; van de Wetering, 2021).

In summary, firms can use EA-driven dynamic capabilities to progress their digital dynamic capability, align business and IT stakeholders and drive innovative ways of working (Korhonen and Halén, 2017; Lumor et al., 2021; van de Wetering, 2021). Furthermore, well-developed EA-driven dynamic capabilities enable firms to combine enterprise-wide IT and business resources flexibly, form the foundation for firms' competitive actions, and release business value (Carugati et al., 2020). This argument is in line with recent work by Li et al. (2022), Törmer and Henningsson (2019b) and Zahoor et al. (2022) that show that dynamic capabilities are crucial in the agile adaption of the firm during the COVID-19 disruptions and the process of levering digital technologies to drive and promote new services.

Based on the preceding, the following is defined:

Hypothesis 1 EA-driven dynamic capabilities enable the development of the firm’s digital dynamic capability.

### 3.2 Digital dynamic capability and its impact on operational digital ambidexterity

Dynamic capabilities are still relevant in stable environments where external changes are seemingly predictable and incremental (Fainshmidt et al., 2019; Lin and Wu, 2014). However, these days, the macro economy’s environmental changes arise more frequently, faster, and become unpredictable and even discontinuous (Li et al., 2022; Ozanne et al., 2022; Wiggins and Ruefli, 2005). Hence, under these circumstances, firms require frequent dynamic capabilities to counterbalance the existing modus operandi, erode quickly, and maintain a competitive edge (Helfat and Peteraf, 2009; Teece, 2007; Zahoor et al., 2022). Firms' digital innovation processes are highly embedded within an environmental context (Chirumalla, 2021; Kohli and Melville, 2019). Previous literature argued that firms could influence the external environment over an extended period. Therefore, the firm’s dynamic capabilities are seemingly context-dependent (Bitencourt et al., 2020; Drnevich and Kriauciunas, 2011). The COVID-19 outbreak forcefully shows its aggravation in a seemingly hyper-competitive and connected world. Under these rapidly changing conditions, firms need to renew themselves and adapt to the new environment while embedding new or enhanced existing capabilities and resources and aligning internal mechanisms and the external environment (Li et al., 2022; Pettus et al., 2009; Sirmon et al., 2007). Therefore, moderns firms must create business value for their customers and external parties (e.g., partners and other collaborators) by exploiting their digital capabilities to effectively use their technical and business resources (Blackburn et al., 2020; Chaudhuri et al., 2022).

Hence, this study argues that digital dynamic capability leads to operational digital ambidexterity. First, a digital dynamic capability is essential to achieve innovation and sustainable improvements at the operational capability level (Khin and Ho, 2019; Wu et al., 2020). This argument is consistent with Wu et al. (2019), who show that such a capability can reinforce existing operations and rapidly develop new digital operational approaches, solutions, and products. Moreover, a digital dynamic capability enables agility in customer-related processes and services by exploiting the firm’s knowledge processes and assets (Ghosh et al., 2021; Lee et al., 2015; Pattij et al., 2022; Shen et al., 2021; Van de Wetering and Versendaal, 2021).

Therefore, this capability supports the organization in learning from experiences during tumultuous times. Hence, under these conditions, firms must search continuously, identify, and absorb new digital innovations such that they can be used to improve their operational capabilities, implement radical, innovative digital technologies, and efficient ways of working (Acur et al., 2010). Hence, operational capabilities depend on the firm’s ability to manage new digital technologies (Khin and Ho, 2019; Nousopoulou et al., 2022; Setia et al., 2013). Also, firms need to deal with the rapidly evolving digital landscape and must therefore have a clear view and vision of their ability to master digital technologies as well as to deploy them in practice (Ghosh et al., 2021; Lee et al., 2015; Ross et al., 2019).

Modern firms need a strong digital dynamic capability to deploy complementary IT resources and align this with what is needed from the business side to gain business value and a competitive edge (Ghosh et al., 2021). Therefore, it is likely that firms that consciously invest and develop such a digital dynamic capability are more likely to develop operational capabilities and achieve operational digital ambidexterity using digital innovations and further generate business value according to the dynamic capability perspective (Khin and Ho, 2019). Therefore, such a significant dynamic capability is essential for firms that focus on improving operational capabilities that serve as a basis for business value. Thus, this study defines:

Hypothesis 2 Digital dynamic capability positively impacts operational digital ambidexterity.

### 3.3 Operational digital ambidexterity and business value

Comprehending customers' behavior and anticipating their needs is critical in the current turbulent market, as COVID-19 has resulted in great uncertainty and an involuntary shift in consumer and b2b wishes, needs, and behavior (Ozanne et al., 2022). Moreover, Papadopoulos et al. (2020) argue that executives and managers should embrace an ambidextrous approach to deploying a digital solution to create business value. However, achieving an ambidextrous orientation in practice is not easy, as the seemingly opposing forces of exploitation and exploration compete for the same firm resources and, therefore, place conflicting demands on the firm (March, 1991; Raisch et al., 2009). Nevertheless, new innovative digital technologies support modern firms to adapt to turbulent conditions (Hanelt et al., 2021; Kraus et al., 2022). Even more so, Nwankpa and Datta (2017) argue that firms' investments in emerging digital technologies will shape their future.

An operational digital exploration capability drives creative ideas and enables firms to change or invent new business operations fundamentally (e.g., product/service development and production, supply chain management, customer delivery) to develop innovative processes and work procedures for daily tasks using digital technologies (Lee et al., 2015). Moreover, as these digital innovations (e.g., analytics, big data, cloud, social media, mobile) are implemented at the operational level, they are tough to replicate by other firms. Also, Li et al. (2013), argue that such a capability enhances new product performance and delivers high-quality products and services. Finally, this operational capability promotes a more resilient reaction to customers' demands and technological changes (Patel et al., 2012).

On the other hand, an operational digital exploitation capability tries to take advantage of new digital solutions to drive continuous adaptions (Davenport, 1993; Lee et al., 2015). This capability enables firms to enhance the efficiency of their current business processes and operations incrementally using digital innovations in a novel way (Raisch et al., 2009). So, firms that foster operational digital ambidexterity under the current COVID-19 shock will implement comprehensive and radical innovative digital technologies in their day-to-day business context. Consequently, these ambidextrous firms can leverage the efficiency within existing business operations by using new innovative digital technologies to lower costs, improve delivery speed and reliability, and customize products and services to suit individual customers (Hwang et al., 2015; Sahi et al., 2020). As a result, firms that can achieve high business value from intermediate-process level impacts can enhance their competitive advantage in the business ecosystem through, e.g., market growth, market share, and increasing revenues (Rodrigues et al., 2020).

In summary, the literature argues that synchronizing or balancing operational digital exploitation capability and operational digital exploration capability will improve efficiency, reduce costs and deliver outstanding and reliable services to customers and thus enhance business value in highly turbulent environments and under uncertain conditions, like under the COVID-19 shock (Patel et al., 2012; Sahi et al., 2020). Firms that lack well-synchronized ambidextrous capabilities typically over-rely on continuous, incremental operational innovations, not appropriately addressing environmental demands (Belhadi et al., 2021; Jansen et al., 2012; Lee et al., 2015; Yang, 2021). Based on the above, this study defines the following:

Hypothesis 3 Operational digital ambidexterity will be positively related to business value under COVID-19.

## 4 Methodology

### 4.1 Sample

An online survey was developed and pretested by three Master students and four senior practitioners. After that, the survey was conveniently administered to possible informants. Respondent entries were treated confidentially and anonymously. Hence, no reference would be made to an individual or a specific firm. The respondents were assured that the outcomes of this study would only be reported on an aggregate level (Podsakoff et al., 2003). Moreover, respondents could withdraw their survey responses whenever they wanted to. Before commencing the survey, respondents were asked to sign a consent form. Moreover, this study did not request any sensitive and personal data, in line with regulation protocols, the faculty’s research principles, and approved by the Research Ethics Committee of the University. The Committee operates under the respective deans of the various Faculties of the University.

This study targeted senior executives and business and IT managers. Students of a strategic IS and architecture course (in total 212) at a Dutch University were asked to participate. These students were also asked to distribute the survey in their professional networks and invite two other professionals familiar with the content.

Finally, the data were conveniently collected between October 12th, to November 20th, 2020. A convenient sampling approach is relatively inexpensive, and a non-random approach to get adequate data from a specific type of respondent that is relatively easy to contact (Etikan et al., 2016). The administrative survey records show that a total of 715 respondents started with the online survey. Of this total amount of unique visitors, 414 entries and cases were finally selected as suitable for subsequent analyses after carefully removing unreliable or incomplete entries. The students are all experienced professionals. Hence, 56% of the respondents showed that they have working experience of at least 11 years. More than a fifth of the total respondents (22%) had more than 25 years of working experience. In total, 55% of all participating respondents were C-level decision-makers (e.g., CEOs, CDOs, CIOs) and business and IT managers. Another large group comprises the firm’s enterprise architects (25%) and IT/business consultants, who comprise 21% of the sample. Most firms were more than 25 years old (i.e., 67%), 8% between 0 and 5 years, 6% between 6 and 10 years old, 12% between 11 and 20, and 7% between 20 and 25 years old. The majority of the respondents (64%) come from the private sector, whereas the public sector accounts for 27%. The respondents' demographics are shown in Table 2.

**Table 2:** Demographics of the respondents.

Items	Categories	*#*	%
Amount of current employees	<100	72	17%
	101–300	35	8%
	301–1000	64	15%
	1001–3000	69	17%
	>3000	174	42%
Function within organization	CEO	17	4%
	CIO/CDO	36	8%
	Manager operations	52	13%
	Innovation manager	27	7%
	Business manager	38	9%
	Business & IT consultant	85	21%
	IT managers	56	14%
	Enterprise architects	103	25%
Sector/industry	Real estate industry	2	0,5%
	Education sector	15	3,6%
	Energy and utilities	18	4,3%
	Telcom industry	8	1,9%
	Technology	61	14,7%
	Local government	19	4,6%
	Government	37	8,9%
	Retail service industry	19	4,6%
	Oil ​and ​gas ​(O&G)	3	0,7%
	Manufacturing	16	3,9%
	Automotive	1	0,2%
	Industrials industry	3	0,7%
	Recruiting services	2	0,5%
	Restaurant industry	6	1,4%
	Hotel industry	6	1,4%
	Defense industry	2	0,5%
	Food and agriculture sector	5	1,2%
	Finance and insurance	81	19,6%
	Mining, metals, chemicals, and paper sectors	4	1,0%
	Consumer business	9	2,2%
	Healthcare	18	4,3%
	Pharmaceutical industry	6	1,4%
	Publishing industry	1	0,2%
	Transportation and logistics	20	4,8%
	Legal industry and law	2	0,5%
	Advisory services	44	10,6%
	Other industry	6	1,4%

### 4.2 Survey development, constructs, and items

This study attempted to include empirical and validated work on selecting measurement scales. Therefore, *EA-driven dynamic capabilities* were measured through the conceptualization of van de Wetering (2021) and van de Wetering et al., 2021a, van de Wetering et al., 2021b and thus, through a reflective-formative type II model (Petter et al., 2007; Sarstedt et al., 2019). This means that the first-order constructs were all reflective, whereas the second-order model was formative. EA-driven dynamic capabilities were operationalized using sixteen indicators, i.e., five measures for sensing (Sense), five measures for mobilizing (Mob), and six for transformation (Trans).

This study uses the item-level interaction (or multiplication) terms of operational digital exploration capability (Opexplore) and operational digital exploitation capability (Opexploit) to operationalize operational digital ambidexterity (Chi et al., 2017; Lee et al., 2015). The measurement items were adopted from Lee et al. (2015). A representative operational digital exploration capability item is implementing radical, innovative digital technologies (e.g., big data, analytics, social media, cloud, and mobile) in business operations. An item for operational digital exploitation capability is improving the efficiency of existing business operations using innovative digital technologies. Respondents were asked to rate relative to other firms in the industry.

Three measures were adopted from Khin and Ho (2019) for the digital dynamic capability (DDC). Respondents were asked to assess the firm’s capabilities in identifying digital opportunities, acquiring important digital technologies, and mastering state-of-the-art digital technologies.

Business value concerns value or the benefits of operational digital ambidexterity supported by the firm’s dynamic capabilities. It was operationalized using four indicators addressing low total quality costs relative to the total output, delivery speed, and reliability, quality of products/services, and customization to suit individual customers in comparison to competitors over the past months (Hsu, 2013; Rodrigues et al., 2020; Van de Wetering, 2019). Also, for these questions, the respondents were asked to rate relatively to competitors in the same industry (for the past few months).

For this work, a seven-point Likert scale was used for all survey items (1: strongly disagree to 7: strongly agree). All items are included in the Appendix – Survey items.

#### 4.3 Data quality assessments and procedures

Data quality assessment is essential before the structural model can be analyzed. Therefore, common method bias (CMB) was assessed through various methods (Podsakoff et al., 2003). First, non-response bias was accounted for by using a T-test comparing responses from early and late respondents. Differences were all non-significant. Also, Harman’s single-factor assessment demonstrated that no single factor was responsible for all the variance. Based on the above analyses and outcomes, it can be concluded that CMB was not a problem (Podsakoff et al., 2003).

Finally, a marker-value was included in the model to check for CMB (Schilke et al., 2009). Hence, a theoretically unrelated variable, i.e., years of business experience (*μ* = 2.82; min = 1, 0–5 years; max = 5, over 25 years; median = 2; Std. = 1.53), was included in the research model. In a subsequent step, it was further analyzed if this marker variable had any significant relationships with (predictor) variables.

The marker variable showed an average correlation of −0.05 with all the model’s constructs. This outcome, once more, shows that CMB is not a problem in this study.

Moreover, the final data sample (*N* = 414) exceeds the minimum requirements (Hair et al., 2017a, Hair et al., 2017b). Therefore, the measurement model can now be subjected to reliability and validity tests.

The measurement model was evaluated using various quality metrics for the first-order reflective constructs. These included the internal consistency reliability (i.e., Cronbach’s alpha, CA) and the composite reliability metric (CR). Also, the average variance extracted (AVE) was analyzed to assess convergent validity (Ringle et al., 2022). The first outcomes of the measurement model analyses reveal that all CA and CR values have at least a value of 0.7. Moreover, the loadings of each item to each construct were all above 0.7. Therefore, the established average variance extracted (AVE) values for the latent constructs exceed the threshold of 0.50 (Hair et al., 2016). Thus, there is sufficient convergent validity of the first-order latent constructs (Ringle et al., 2022).

Three complementary methods were used to assess the degree to which each construct (that should not relate to other constructs) is unrelated based on empirical validation, i.e., discriminant validity. As a first step, it was assessed if cross-loadings of all construct items loaded more strongly on their own construct than on other constructs. As a result, all items loaded more strongly on their construct. Secondly, this study compared the square root of the AVE (i.e., Fornell-Larcker criterion) with correlating values on other constructs, i.e., a cross-correlating analysis (Hair et al., 2016). The cross-correlating values should not be larger than this Fornell-Larcker criterion to establish discriminant validity (Hair et al., 2016), which is the case in this current work. The heterotrait-monotrait ratio of correlations (HTMT) value was used as a final method to identify possible discriminant validity issues (Hair et al., 2017a, Hair et al., 2017b). This relatively new criterion is considered a better approach than these two metrics to establish discriminant validity in PLS-SEM studies (Hair et al., 2017a, Hair et al., 2017b). Furthermore, outcomes showed that all the correlating values are below the threshold value of 0.85. These outcomes imply that discriminant validity is established. Finally, no multicollinearity could be detected within the current data. Hence, variance inflation factors (VIFs) values for the second-order construct, EA-driven dynamic capabilities (the reflective-formative type II model), all were substantially lower than the threshold value of 3.5 (Hair et al., 2019).

## 5 Results

The hypotheses were tested using PLS-SEM, which is a variance-based approach to SEM rather than covariance (Dash and Paul, 2021; Hair et al., 2016). This approach is best suited for this current research as the research model includes both formative and reflective (higher-order) constructs (Hair et al., 2017a, Hair et al., 2017b; Riemenschneider and Armstrong, 2021), and the focus of the analyses is prediction-oriented (Hair et al., 2016, 2017a, 2017b). Moreover, PLS-SEM estimates both the measurement and structural model, and the algorithm tries to maximize the variance explained in dependent constructs and variables as part of a complex multiple mediation value path (Dash and Paul, 2021; Petter, 2018).

**Figure 2:**
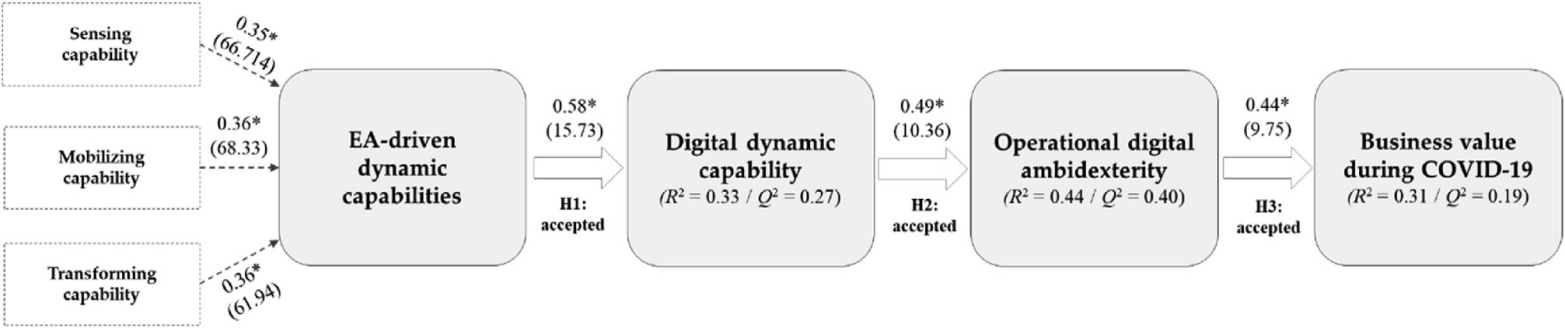
Summary of the structural model results.

This study used the tool SmartPLS v. 4.0.8.4 to run the PLS algorithms and analyses (Ringle et al., 2022), and for the bootstrapping de defacto, 5000 subsamples were applied (Hair et al., 2016). The coefficient of determination (*R*^2^) is the measure to assess the PLS algorithm’s predictivity. The results of the structural model analyses are summarized in Figure 2.

Hence, it shows the hypothesized value paths, regression coefficients (Beta’s), T-values, the *R*^2^, and their associated predictive values Stone-Geisser’s *Q*^2^ value (i.e., a criterion of predictive relevancy). Based on these outcomes, it can be concluded that the hypothesized relationships can all be confirmed. Furthermore, in these analyses, the value path was controlled for the (non-significant) effect of the ‘age of the firm’ (*β* = −0.031, *t* = 0.71, *p* = .48).

Lastly, this study used a blindfolding procedure, i.e., a sample reuse method, to assess the predictive relevance of this study’s model (Hair et al., 2019). The blindfolding results, interpreted through the Stone-Geisser index (*Q*^2^) for all dependent constructs, are all beyond 0, showcasing the model’s predictive sufficiency.

### 5.1 Multiple mediation analyses

This study followed specific mediation guidelines (Ferreras-Méndez et al., 2021; Hair et al., 2016) to investigate the research model’s imposed mediating value paths. As such, the direct structural contribution of dynamic capabilities on operational digital ambidexterity is significant (β = 0.53, t = 15.845, p ≤ 0.0001). Hayes (2013) argues that this outcome is crucial for assessing mediation. The procedure’s next step is to test whether or not the mediating paths are significant. Therefore, this path, including digital dynamic capability, was subsequently run using the PLS software. Outcomes of the analyses show that the direct, structural relationship, i.e., dynamic capabilities → operational digital ambidexterity, is significant, although not as strong when the digital dynamic capability was excluded from the model (β = 0.26, t = 5.42, p = 0.26). These outcomes suggest that there is a complementary partial mediating relationship. Next, the model implies that operational digital ambidexterity is a mediating construct in the value path (i.e., mediating the impact of digital dynamic capability). A similar approach was used to test this part of the research model. Hence, outcomes showcase that operational digital ambidexterity fully mediates digital dynamic capability on business value. Hence, digital dynamic capability’s direct effect on business value showed a significant relationship (β = 0.41, t = 5.42, p ≤ 0.0001). When operational digital ambidexterity was included as a mediating force, this relationship became insignificant (β = 0.01, t = 1.72, p = 0.08), while operational digital ambidexterity’s effect on business value was significant (β = 0.49, t = 9.95, p ≤ 0.0001). Table 3 also includes specific indirect effects.

**Table 3:** Results of the structural model assessment, including effect sizes.

Specified path	Effect	Effect size (*f*^2^)	Bias-corrected confidence interval	*t*-value	Significant	Outcome
DC → BV	0.09	0.01	CI (-0.074–0.2147)	1.84	NO	No direct effect
DC → DDC	0.58	0.49	CI (0.50–0.64)	15.73	YES	H1 Supported
DDC → ODA	0.49	0.28	CI (0.39–0.57)	10.36	YES	H2 Supported
ODA → BV	0.44	0.17	CI (0.40–0.59)	9.75	YES	H3 Supported
DDC → BV	0.07	0.00	CI (-0.051 – 0.187)	1.21	NO	Not significant
DC → DDC → ODA (*Mediation by DDC*)	0.28	-	CI (0.22–0.35)	8.42	YES	
DDC → ODA → BV (*Mediation by ODA*)	0.22	-	CI (0.16–0.30)	6.52	YES	
Age → BV	-0.031	-0.005	CI (-0.12 – 0.005)	0.71	NO	No confounding

*Note:* DC—Dynamic capabilities; DDC—digital dynamic capability; ODA—operational digital ambidexterity; BV—business value

## 6 Discussion

Under the current COVID-19 shock, it is needless to say that firms that are dynamically capable and effective with their operational digital innovations are better equipped to deal with uncertainty and deliver outstanding and reliable services to customers. However, notwithstanding the valuable contributions to digital capability-building, it remains unclear how firms can leverage their digital initiatives to achieve operational excellence and digital transformations using EA-driven dynamic capabilities (Shen et al., 2021; van de Wetering et al., 2021a, 2021b). Thus, the associations between dynamic capabilities driven by EA, digital-driven benefits, and business value have neither been empirically investigated nor statistically estimated. Hence, this field of study lacks a clear theoretical foundation (Clauss et al., 2022; van de Wetering, 2021). Although limited in focus, this study tried to bridge this current gap. Following the DCV, this study developed a research model and empirically tested the underlying hypotheses using survey data from 414 senior professionals. As a result, support was found for each of the hypotheses.

This study takes a different lens in conceptualizing dynamic capabilities than previous information systems and management studies (Drnevich and Kriauciunas, 2011; Loureiro et al., 2021; Protogerou et al., 2012; Randhawa et al., 2021; Teece, 2007). Specifically, it is argued that EAs can be leveraged within firms, but only when they are infused in their dynamic capabilities. Therefore, this study conceived ‘EA-driven dynamic capabilities’ as dynamic capabilities. These EA-driven capabilities allow firms to continuously sense the ongoing firm-wide transformations in turbulent times and adequately respond to developments by orchestrating their business and IT resources and driving their digital capabilities (Shanks et al., 2018; van de Wetering, 2021; van de Wetering et al., 2021a, 2021b).

Based on the micro-foundations and conceptualization of dynamic capabilities (Teece, 2007) and recent EA-capabilities studies (Shanks et al., 2018; Törmer and Henningsson, 2019b; van de Wetering et al., 2021a, 2021b; van de Wetering et al., 2020), three unique capabilities can be distinguished. These are (1) a ‘Sensing’ capability, (2) a ‘Mobilizing,’ and finally, (3) a ‘Transforming’ capability. These three EA-driven dynamic capabilities can be used to deliver business value to firms and thrive under the COVID-19 pandemic.

EA-driven dynamic capabilities positively influence digital dynamic capability, which extends previous studies' findings (Shanks et al., 2018; van de Wetering, 2021). Previous work showed that dynamic capabilities driven by EA are essential for developing digital dynamic capability and the firm’s digital transformations, but empirical support was lacking (Gong et al., 2020; Korhonen and Halén, 2017). Therefore, this current study contributes to the much-needed theoretical foundations of EA-value creation, empirically showing that EA-driven dynamic capabilities are a key antecedent of digital dynamic capability. Thus, EA-driven dynamic capabilities are crucial for a firm to manage innovative digital technologies like big data analytics, AI, and IoT for exceptional and effective service development.

The second result of our work was that digital dynamic capability positively impacts operational digital ambidexterity. This key finding extends the work by Chaudhuri et al. (2022), Khin and Ho (2019) and Van de Wetering and Versendaal (2021), who argued that such a capability is a driver of operational capabilities and efficient and agile ways of working. Furthermore, this work now shows that digital dynamic capability equips firms with the ability to deal with the rapidly evolving digital landscape and improve operational capabilities through a dual digitalization approach, thus, continually innovating and improving the firm’s operational processes using digital technologies through an exploratory mode and an exploitation mode.

The third key result is that this study provides empirical evidence that operational digital ambidexterity positively influences business value under COVID-19. Previous work showed that an ambidextrous IT-practice could profoundly impact firms' balanced pursuit of operational exploration and exploitation ambidexterity (Lee et al., 2015). This study shows that firms fostering operational digital ambidexterity under the current COVID-19 shock can leverage efficiency within existing business operations by using new digital technologies. Hence, these firms are better equipped to lower costs, improve delivery speed and reliability, and customize products and services to suit individual customers, thus creating business value under COVID-19. This particular finding extends the work of Patel et al. (2012), Rodrigues et al. (2020) and Sahi et al. (2020) on achieving digital-driven business value in highly turbulent environments and under uncertain conditions.

These core contributions extend current insights and fill the gap in the extant literature.

### 7.1 Theoretical contributions

The work makes three contributions to theory. *First*, this study’s ground was built on both the shortcomings and foundations of previous scholarship and is the first that sets forward the notion of EA-driven dynamic capabilities and their indirect impact on the firm’s business value under COVID-19. It shows the mediating role of digital dynamic capability and operational digital ambidexterity in the value path. These results are important as previous scholarship did not fully grasp the value-path of EA-driven capabilities (Shanks et al., 2018; Tamm et al., 2022; Van de Wetering, 2019) and how firms can leverage their dynamic capabilities and digital investments and create business value under turbulent conditions like the COVID-19 shock. The outcomes of this study can, thus, be very an anchor point for scholars to comprehend the role, value, and contributions of dynamic capabilities under tumultuous times (Lin and Wu, 2014; Moreno et al., 2020; Ozanne et al., 2022; Van de Wetering, 2019). These contributions are also significant concerning the IS discipline’s role in addressing the great uncertainty and today’s many complex challenges (Sarker et al., 2019).

Moreover, this work reveals that firms that have developed their dynamic capabilities are well-equipped to drive digital innovations and embed them into daily operations. Consequently, they will become ambidextrous into operational capabilities and gain business value from digital investments. This work’s *second* contribution is the extension of the current EA-driven capabilities body of work by unfolding various types of benefits that can be achieved in the EA-value path (Niemi and Pekkola, 2019; Shanks et al., 2018; Tamm et al., 2022; Van de Wetering, 2019). These insights advance our understanding of how EA delivers value and strengthens the capability-building process for digital transformations. This is important as empirical evidence on this issue remains scarce in the literature (Grave et al., 2021; Marx et al., 2021; Pattij et al., 2022; Van de Wetering, 2019). Moreover, these theoretical contributions are important as scholars could use the results while examining firms' dynamic, digital, and operational capabilities under turbulent conditions.

*Third*, the focal point of the extant literature on dynamic capabilities has been how modern firms respond to environmental turbulence by enabling managers and decision-makers to create competitive value by modifying capabilities on the operational level (Protogerou et al., 2012; Shanks et al., 2018). The outcomes of this current study extend these scholarly contributions by demonstrating how dynamic capabilities driven by EA support the process of developing the firm’s digital dynamic capability and driving digital ambidexterity on the operational level. Operational digital ambidexterity is a crucial quality that firms need under the current COVID-19 shock to accelerate digitization and achieve business value (Heredia et al., 2022; Lee et al., 2015; Shen et al., 2021; Soto-Acosta, 2020).

### 7.2 Managerial implications

The pressure to compete and deliver immediate results coupled with rapidly changing market dynamics has driven firms to search for new ways to innovate and find solutions. However, the IT and business landscape has changed when these solutions are implemented and deployed. So, to compete effectively requires a solution that creates business value for firms while enabling a sustained ability to change. Therefore, the managerial implications will now be presented.

*First*, the study results suggest that business and IT executives and senior professionals must have a rich overview of the maturity and development of their EA-driven sensing, mobilizing, and transforming capabilities. These capabilities form essential ingredients for firms to survive in crisis situations like COVID-19 and drive the firm’s digital dynamic capability to transform the customer experience, operational processes, and even the firm’s operational functioning. Therefore, it is now argued that appropriate conditions for developing and shaping these capabilities must be created. Developing capabilities takes time. However, decision-makers should focus on capability-building efforts and not drive the competitiveness and fitness of the firm directly; instead, they should drive those capabilities that allow firms to transform operational and business models. This work unfolds the critical role of EA-driven dynamic capabilities and the value they create for firms. Time, effort, and dedication in funding and putting highly skilled multidisciplinary teams to mature these capabilities within the firm further could contribute to this. Firms with superior digital dynamic capability are more likely to become operational digital ambidextrous and achieve higher business value and competitive actions.

*Second*, the concept of EA-driven dynamic capabilities contains several underlying, strategic, and synergetic practices driven by EA. In isolation, investing in each of these elements is unlikely to achieve the desired outcomes (Abbu and Gopalakrishna, 2021; Teo and Ranganathan, 2003). As such, synergistic bundling effects will have a more profound impact than optimizing the capabilities in isolation (Adegbesan, 2009; Li and Jia, 2018). Thus, senior managers should diagnose the current development of the capabilities using the provided indicators and outline an integrated change development program encompassing several initiatives required to drive digital and operational capabilities and achieve high business value.

*Third*, this study shows that EA needs to be actively infused into the firm’s dynamic capabilities to respond to changes adequately. However, it should be noted that EA is an ongoing effort to inform and guide the implementation and alignment of IT that serves the business and its key stakeholders (Carugati et al., 2020; Van Der Raadt, Schouten and Van Vliet, 2008). Therefore, we suggest that the EA practice translates the business and IT strategy into transparent and clear to-be architectures (or blueprints) and consciously develops improvement roadmaps to move systematically from the current situation to a to-be situation and ensure projects deliver following this roadmap. Such an approach will support business priorities and an architecture governance model to promote transparency by adopting a common language amongst stakeholders and prioritizing the IT landscape planning process, considering the most critical business processes and the firm’s dynamic capabilities.

*Fourth*, this study shows that contemporary firms should strive for a dual digital approach to operational capabilities to bring the highest degree of business value. This approach is characterized by developing an exploratory mode, where firms fundamentally change or invent new business operations to create new ways of performing daily tasks using digital technology and an exploitation mode. This mode strives for operational productivity by improving current operations' efficiency and cycle time and reducing costs using digital technology. The outcomes of this work show that firms need to strengthen the development of EA-driven dynamic capabilities to become ambidextrous in digitalization. Hence, this work gives managers the insights to act under changing circumstances while showcasing the digital organizational capabilities that allow firms to deliver products and services now and drive business value.

## 7.3 Limitations and future research

The study’s limitations will now be discussed, as several limitations could provide opportunities for future work. First, the current study used subjective measurement items obtained through the survey. Although these subjective and perceptual measures correlate strongly with objective data (Wall et al., 2004), triangulation with available archival or publicly available data could strengthen the study results.

Second, closely related to the first limitation is that the final data were obtained using a single informant strategy. Although this approach is in line with previous work (Nguyen et al., 2021), method bias might be a concern for this research. Nevertheless, this study accounted for possible measurement errors and method bias, and future work could use a matched-pair approach to remedy possible risks associated with this approach. In such a matched-pair approach, different respondents address independent and dependent constructs of the research model.

Third, this research embraced a convenient sampling approach. Although this approach is suitable for getting data from a targeted group of respondents, there are some limitations to this method that should be acknowledged. Most prominently, this approach assumes that the obtained data sample represents the population, a risky assumption in a non-probability context as this is difficult to assess whether or not this holds (Etikan et al., 2016).

Fourth, the data were gathered from firms in the Netherlands. Future work could replicate this study, possibly in other countries, and see whether the key relationships of the research model hold. Third, the study used a cross-sectional design, collecting data at a specific time. However, a firm’s business value and performance may vary over time. These evolutionary change patterns are worth investigating (Tanriverdi et al., 2010; Walraven, van de Wetering, Helms, Caniëls and Versendaal, 2022) but are currently well beyond the scope of this work.

Future research could also investigate other contextual and organizational conditions relevant to enhancing business value in increasingly complex business environments. Hence, future work could adopt a holistic configurational approach as there are distinctly different approaches to change and transformation depending on the challenge faced by each firm.

## Declarations

### Author contribution statement

Rogier Van de Wetering: Conceived and designed the experiments; Performed the experiments; Analyzed and interpreted the data; Contributed reagents, materials, analysis tools or data; Wrote the paper.

### Funding statement

This research did not receive any specific grant from funding agencies in the public, commercial, or not-for-profit sectors.

### Data availability statement

The authors do not have permission to share data.

### Declaration of interest’s statement

The authors declare no conflict of interest.

### Additional information

No additional information is available for this paper.

## Appendix D Supplementary data

renamed_0b579 renamed_0b579
