# Supplementary material for: The role of enterprise architecture-driven dynamic capabilities and operational digital ambidexterity in driving business value under the COVID-19 shock
Source: Heliyon. 2022 Nov 8;8(11):e11484. doi: 10.1016/j.heliyon.2022.e11484 (PMC9640386; doi:10.1016/j.heliyon.2022.e11484)
Supplement: renamed_0b579 [file mmc1.docx]

**Appendix – Survey items**

**The role of enterprise architecture-driven dynamic capabilities and operational digital ambidexterity in driving business value under the COVID-19 shock**

***Sensing capability***

*Reliability and validity statistics: CA:* *0.90; CR: 0.93; AVE:* *0.77*

SEN1: We use our EA to identify new business opportunities or potential threats

SEN1: We review our EA services (e.g., providing content, EA standards, skills, and knowledge) regularly to ensure that they are in line with what our key (internal and external) stakeholders want

SEN3: We adequately evaluate the effect of changes in the baseline and target EA on the organization

SEN4: We devote sufficient time to enhancing our EA to improve business processes

SEN5: We develop greater reactive and proactive strength in the business domain using our EA

***Mobilizing capability***

*Reliability and validity statistics: CA:* *0.91; CR: 0.93; AVE:* *0.79*

MOB1: We use our EA to draft potential solutions when we sense business opportunities or potential threats

MOB2: We use our EA to evaluate, prioritize and select potential solutions when we sense business opportunities or potential threats

MOB3: We use our EA to mobilize resources in line with a potential solution when we sense business opportunities or potential threats

MOB4: We use our EA to draw up a detailed plan to carry out a potential solution when we sense business opportunities or potential threats

MOB5: We use our EA to review and update our practices in line with renowned business and IT best practices when we sense business opportunities or potential threats

***Transforming capability***

*Reliability and validity statistics: CA:* *0.90; CR: 0.93; AVE:* *0.78*

TRS1: Our EA enables us to successfully reconfigure business processes and the technology landscape to come up with new or more productive assets

TRS2: We successfully use our EA to adjust our business processes and the technology landscape in response to competitive strategic moves or market opportunities

TRS3: We successfully use our EA to engage in resource recombination to match our product-market areas and our assets better

TRS4: Our EA enables flexible adaptation of human resources, processes, or the technology landscape that leads to competitive advantage

TRS5: We successfully use our EA to create new or substantially changed ways of achieving our targets and objectives

TRS6: Our EA facilitates us to adjust for and respond to unexpected changes

***Digital dynamic capability***

*Reliability and validity statistics: CA:* *0.87; CR: 0.91; AVE:* *0.81*

DDD1: Acquiring important digital technologies

DDD2: Identifying new digital opportunities

DDD3: Mastering the state-of-the-art digital technologies

***Operational digital exploration capability***

*Reliability and validity statistics: CA:* *0.90; CR: 0.93; AVE:* *0.84*

ODEXL1: Implement extensive innovative digital technologies (e.g., analytics, big data, cloud, social media, mobile) in business operations (e.g., product/service development and production, supply chain management, customer delivery, employee management)

ODEXL2: Implement radical innovative digital technologies in business operations

ODEXL3: Implement operational innovative digital technologies that are difficult to replicate by other firms

***Operational digital exploitation capability***

*Reliability and validity statistics: CA:* *0.91; CR: 0.94; AVE:* *0.88*

ODEXT1: Reduce the cost of existing business operations using innovative digital technologies (e.g., analytics, big data, cloud, social media, mobile)

ODEXT2:Improve the cycle time of existing business operations using innovative digital technologies

ODEXT3:Improve the efficiency of existing business operations using innovative digital technologies

***Business value***

*Reliability and validity statistics: CA:* *0.72; CR: 0.83; AVE:* *0.56*

BV1: Has very low total quality costs relative to the total output

BV2: Reveals outstanding delivery speed and reliability

BV3: Delivers high-quality products/services

BV4: Customizes products and services to suit individual customers
